# Short-term changes in chest CT images among individuals at low altitude after entering high-altitude environments

**DOI:** 10.3389/fpubh.2024.1392696

**Published:** 2024-07-01

**Authors:** Peng Wang, Zhiwei Zuo, Jie Wu, Jianxiong Wang, Rui Jiang, Feizhou Du

**Affiliations:** ^1^Department of Radiology, The General Hospital of Western Theater Command, Chengdu, China; ^2^Department of Radiology, Tibet Ali Prefecture People's Hospital, Tibet, China

**Keywords:** plateau, plateau settlers, plateau environment acclimatization, spontaneous pneumomediastinum, spinal epidural space, multiple interstitial emphysema

## Abstract

**Objective:**

To investigate the short-term changes in chest CT images of low-altitude populations after entering a high-altitude environment.

**Methods:**

Chest CT images of 3,587 people from low-altitude areas were obtained within one month of entering a high-altitude environment. Abnormal CT features and clinical symptoms were analyzed.

**Results:**

Besides acute high-altitude pulmonary edema, the incidence of soft tissue space pneumatosis was significantly higher than that in low-altitude areas. Pneumatosis was observed in the mediastinum, cervical muscle space, abdominal cavity, and spinal cord epidural space, especially the mediastinum.

**Conclusion:**

In addition to acute high-altitude pulmonary edema, spontaneous mediastinal emphysema often occurs when individuals in low-altitude areas adapt to the high-altitude environment of cold, low-pressure, and hypoxia. When the gas escapes to the abdominal cavity, it is easy to be misdiagnosed as gastrointestinal perforation. It is also not uncommon for gas accumulation to escape into the epidural space of the spinal cord. The phenomenon of gas diffusion into distant tissue space and the mechanism of gas escape needs to be further studied.

## Introduction

The decrease in oxygen partial pressure with increasing altitude is a well-known phenomenon. Individuals accustomed to living at low altitudes who suddenly find themselves at high-altitude experience a reduction in the diffused oxygen through their alveoli, leading to a decrease in oxygen saturation. As a result of this hypoxia, people commonly experience various physiological reactions, including headache, dizziness, nausea, vomiting, palpitations, and shortness of breath (1). When individuals present with chest discomfort, chest CT examination is the preferred imaging method as it can accurately depict structural abnormalities in the heart and lungs. While it is generally accepted that changes in the heart and lungs at high-altitude occur due to chronic high-altitude adaptation, only a few isolated cases have been reported during the early stages of this process (2, 3). Moreover, it is mostly related to aviation flight (4–7). Therefore, we aim to conduct a comprehensive cross-sectional survey to investigate whether there are abnormal changes in CT images and to assess the extent of chest discomfort during the early phase of acclimatization to the plateau environment.

## Materials and methods

Data collection was conducted at Ali District People’s Hospital (located at an altitude of 3,670 meters) from January 2019 to May 2021. The retrospective study received approval from The Ethics Committee of The General Hospital of Western Theater Command Hospital. The written informed consent from the patients was waived. All the methods were carried out in accordance with relevant guidelines and regulations to ensure full patient and family understanding of the experimental procedures and their rights.

Inclusion criteria: (1) Migrant workers and tourists residing in low-altitude areas in central and eastern China must be over 18 years of age and have a living environment below 700 meters. (2) The first chest CT examination was performed within 1 month after arrival at the plateau. (3) With no history of cardiopulmonary disease and chest trauma.

Exclusion criteria: (1) Inadequate image quality for accurate diagnosis. (2) Patients who discontinued their participation during the study.

We also collected baseline data such as gender, age, and symptoms of patients.

## Image acquisition and image diagnosis

All patients completed an outpatient medical history collection, inquiry, and physical examination. A chest CT scan was performed using a German Siemens SOMATOM Definition AS 64-slice CT scanner. The scanning range extended from the upper edge of T1 to the lower edge of the L1 spinous process. Parameters for the scan were as follows: (1) Tube voltage was usually set to 120 kV, but for patients with a thin body type, it could be set to 100 kV. For patients with an obese body type, it could be set between 120 and 140 kV (2). Tube current was automatically regulated to ensure that CTDlvol was less than 4 mGy (3). The display field (DFOV) was set to 33–35 cm (4). The acquisition method used was volume acquisition with an acquisition layer thickness of 5 mm and an acquisition interval of 5 mm. Additionally, conventionally reconstructed 1–1.25 mm thin layer images were transmitted to PACS and workstations for backup (5). The reconstruction algorithm used for the lung window was the lung algorithm with a convolution kernel B50, while the standard algorithm was used for the mediastinal window (soft tissue window).

To evaluate the presence or absence of pulmonary exudative lesions, pneumothorax, pleural effusion, cardiac morphology, mediastinum, and chest wall structure, two radiologists with more than 5 years of experience double-blindly read and scored the above indicators.

## Result

The study was approved by the ethics committee of our hospital. We retrospectively analyzed the chest CT examination data of 3,587 subjects (3,007 males, age 19 ~ 56, average age ± SD = 35.40 ± 10.29) from January 2019 to May 2021 admitted to a medical station located in Ali, Tibet. This hospital is located at an elevation of about 3,670 meters, its service regions range from 3,000 to 5,400 meters, and the annual average temperature of Ali was −9°C. Out of a total of 3,587 cases, 94 individuals showed spontaneous pneumatosis (SP) in various tissue spaces, representing 2.62% (94 out of 3,587) of the total cases. The manifestations included subcutaneous emphysema (SE) in the neck, face, trunk, intra-vertebral canal, and pneumomediastinum (SPM).

The individuals with SP included in our study were 19–51 years old (average age = 29.36, SD = 7.41), and among them, 9 cases ≤20-year-old, 40 cases were 21–30-year-old, 23 cases were 31–40-year-old, 16 cases were 41–50-year-old, and 6 cases >50-year-old. Seventy-seven of 94 cases were male (77/94, 81.91%). The duration of these individuals stayed at Ali was as follows: 22 cases <2 weeks, 36 cases were 2–4 weeks, 25 cases were 4–8 weeks, and 11 cases were >8 weeks. No obvious predisposing cause was found in 90 cases (90/94, 95.74%), and 1 case with fever (1/94, 1.06%), 3 cases with trauma (3/94, 3.19%). The clinical manifestations are shown in Table 1.

**Table 1:** Main clinical manifestations in patients with spontaneous pneumatosis.

Clinical features	Frequency	Proportion (%)
Negative	21	22.3
Cough	16	17
Dyspnea	13	13.8
Chest pain	13	13.8
Oppression in chest	12	12.8
Pharyngodynia	9	9.6
Epigastric pain	5	5.3
Palpitation	3	3.2
Hyperpyrexia with chills	1	1.1
Vertigo	1	1.1

**Figure 1:**
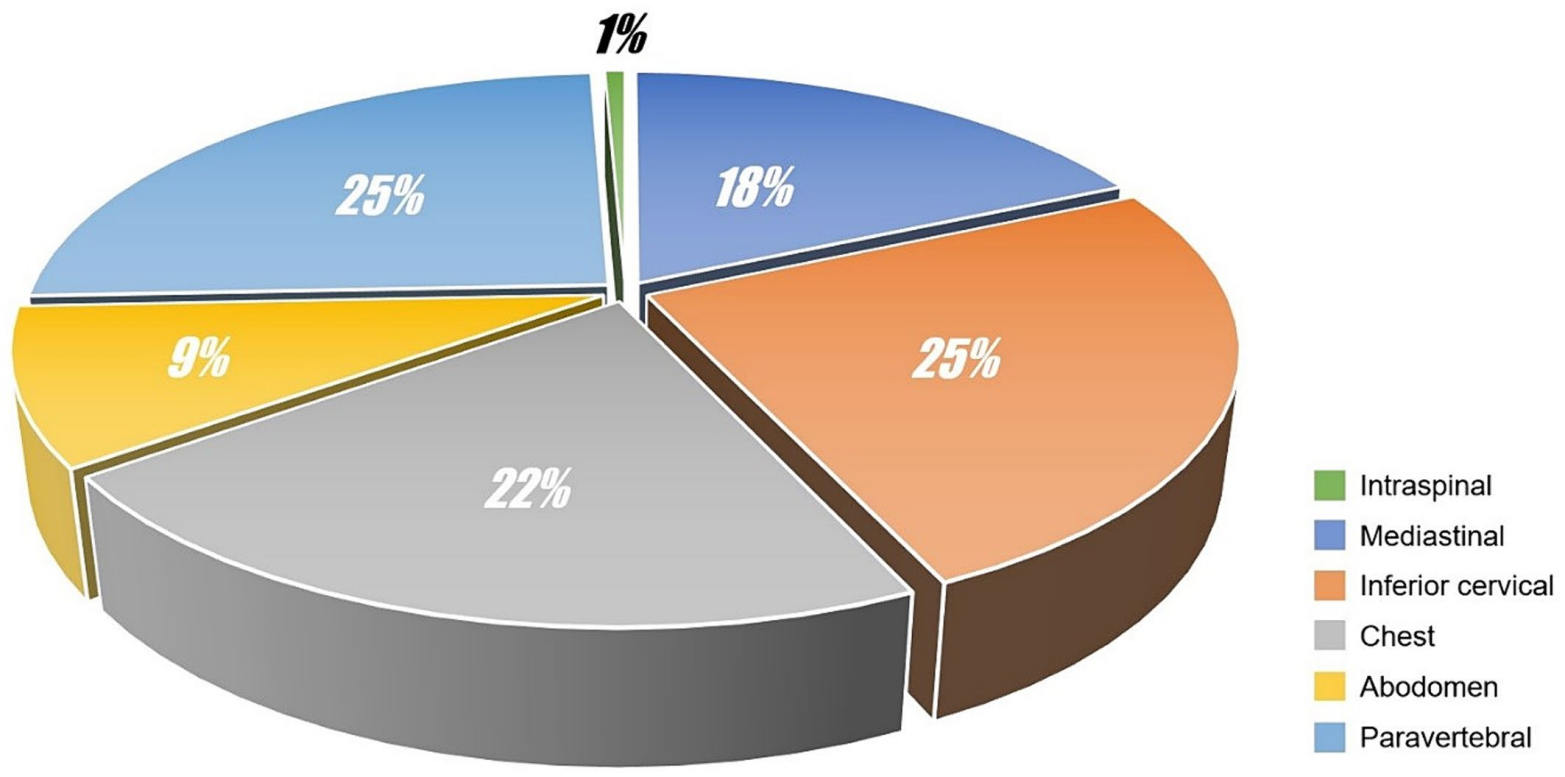
Location distribution of spontaneous pneumatosis.

For each case, the SP was independently evaluated by 2 radiologists (work experiences of whom were 21 years and 17 years respectively). The volume of SP was graded according to the following criteria: (i) mild pneumatosis, the short diameter of maximum cross-section ≤2 mm or the number of independent pneumatosis ≤5; (ii) moderate pneumatosis, the short diameter of maximum cross-section >2 mm and ≤5 mm or the number of independent pneumatosis >5 and ≤10; (iii) severe pneumatosis, the short diameter of maximum cross-section >5 mm or the number of independent pneumatosis >10. Possible disagreements were solved by consensus. The diagnosis results were as follows: 40 cases (40/94, 42.55%) were mild pneumatosis, 24 cases (24/94, 25.53%) were moderate pneumatosis and 30 cases (30/94, 31.91%) were severe pneumatosis. The locations of SP were also concluded (see Figure 1). In addition to SP, we also identified 3 cases with pneumon-edema (3/94, 3.19%), 3 cases with pulmonary bulla (3/94, 3.19%), 1 case with pneumonia (1/94, 1.06%), and 87 cases with negative CT signs in lungs (87/94, 92.55%). Moreover, subphrenic-free air was found in 14 cases (14/94, 14.89%), and 9 cases (9/14, 64.29%) were misdiagnosed as gastrointestinal perforation.

## Discussion

Plateau and alpine areas above 3,000 meters in western China comprise approximately one-sixth of China’s total land area. With the recent development of western China, there has been an increase in the construction of engineering projects and the growth of the tourism economy in these regions. Consequently, a significant number of people from lower altitudes are now traveling to the western high-altitude regions.

In addition to pulmonary edema, brain edema, and other common high-altitude-related diseases, the incidence of subcutaneous emphysema (SE) is also significantly higher in high-altitude areas compared to low-altitude areas. This may be attributed to the low temperature, low pressure, and hypoxic conditions prevalent in high-altitude environments. To the best of our knowledge, this is the first cross-sectional report presenting a large amount of data on SP affecting multiple tissue spaces at high-altitude.

Most SP cases involve young and middle-aged males, primarily engineers and tourists who have recently arrived in highland areas from lowland regions (with an adaptation time of less than 3 months), soldiers, and a few local residents. Due to the extreme natural environment and challenging conditions, the older adult, weak individuals, women, and children rarely venture into the plateau. Our data suggests that the incidence of SP affecting multiple tissue spaces during the altitude acclimatization period is significantly higher than that observed in a stable lowland environment.

We hypothesize that these subcutaneous emphysemas (SEs) in various locations may originate from spontaneous pneumomediastinum (SPM). SPM is a rare, self-limiting condition characterized by the presence of free air within the mediastinum without any association with chest trauma, surgery, or underlying diseases. Its incidence ranges from 0.002 to 0.125% (8).

The middle layer of the deep cervical fascia has thin and loose anatomical characteristics. The anterior tracheal fascia forms in front of the trachea, the thyroid pseudocapsule forms in front of the thyroid gland, and the carotid artery is located on both sides. Various fascias, sheaths, and envelopes create gaps, providing the anatomical basis for the development of mediastinal and subcutaneous emphysema. The potential space within the mediastinum is interconnected, and the space surrounding the esophagus and trachea in the mediastinum extends into the potential space around the esophagus and trachea in the neck. It is also connected to the abdominal connective tissue and space through the thoracic rib triangle of the esophagus and lung. These anatomical features of the mediastinum make it susceptible to the development of mediastinal emphysema.

SPM is considered a result of alveolar rupture which generates free air that could flow into the mediastinum along the tunica-vaginalis around the pulmonary vasculature and could be triggered by breath-holding after inhalation and intense cough, and commonly seen in patients with bronchial asthma, bronchiolitis, or pertussis (9, 10). Most cases involved in our study had no clear clinical inducement. The atmospheric pressure at high-altitude is low, and intra- and extra-pulmonary pressure differences are imbalanced, which could result in pressures in some deep alveolar areas being greater than that in tissue space over a short term. Moreover, due to the decrease of oxygen levels at high-altitude, the atmospheric pressure and oxygen partial pressure above 3,000 meters are about 69.51 kPa (522.6 mmHg) and 14.55 kPa (109.4 mmHg) respectively, which are only about 68.76 and 68.81% of these pressures in sea level respectively, and in such environment, the ventilation of the body will be enhanced and the diffusion capacity of the alveolar membrane will be improved.

**Figure 2:**
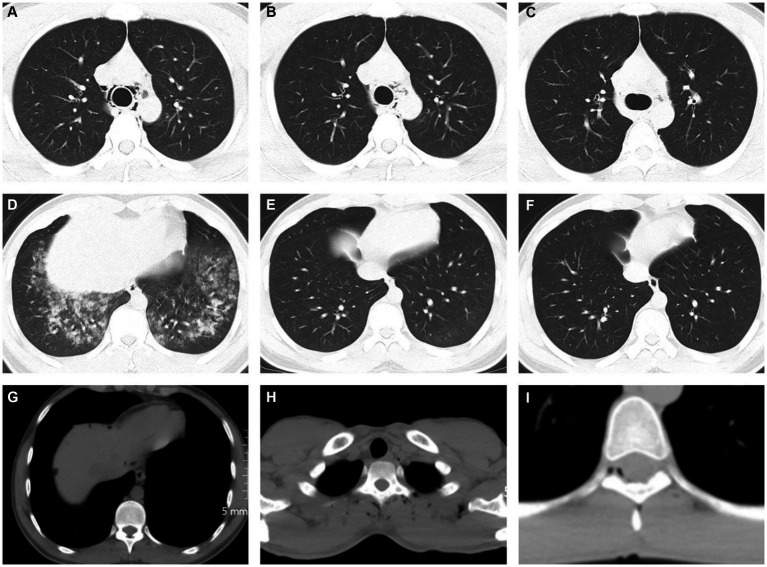
Chest CT images showed spontaneous pneumatosis. **(A–F)** 21 years old, male, **(A,D)** showed a small volume of air in the mediastinum with pulmonary edema and large exudative lesions in both lower lungs, **(B,E)** on day 3, partial absorption of gas accumulation was reexamined, and pulmonary edema was significantly absorbed, **(C,F)** on day 7, pneumatosis and intrapulmonary exudate were reexamined for complete absorption. **(G)** A 25-year-old man presented with a patchy free gas shadow below the diaphragm at the edge of the liver. **(H)** A 23-year-old male with multiple gas accumulation near the spine and left periscapular space. **(I)** A 25-year-old man presents with pitted pneumatosis below the medial margin of the spinal canal.

Extradural intra-vertebral pneumatosis was also observed in our study, as shown in Figure 2. To the best of our knowledge, this is the first study to report extradural intra-vertebral pneumatosis at high-altitude. Another noteworthy finding was the high proportion of SP cases accompanied by free intraperitoneal air, which is not commonly observed at low altitudes. This phenomenon is likely related to the management of dyspnea. Dyspnea is a common symptom experienced at high-altitude, and one of the conventional treatments for dyspnea is lying down. The anatomical structure of the abdomen is relatively less compact, allowing for the diffusion of air in tissue spaces when in a supine position. This diffusion may not necessarily be directed toward the neck or shoulders, but can instead move downwards to the abdomen through the diaphragmatic hiatus. Due to a lack of understanding of SP in different tissue spaces in primary healthcare settings, misdiagnoses can occur. For example, in our study, 9 cases with subphrenic free air were initially misdiagnosed as gastrointestinal perforation, resulting in 5 patients receiving incorrect treatment for gastrointestinal perforation in the clinic. Additionally, we identified 3 cases of SPs in the paravertebral interosseous space (Figure 1), which did not resolve or decrease after 7 days. However, SPM alone cannot fully explain these observed signs. We speculate that factors related to the plateau environment and the composition of the gas within the pneumatosis may play critical roles in the occurrence of SP in these rare locations.

It is important to highlight that high-altitude can lead to the development of spontaneous pneumatosis (SP) in different tissue spaces, including pneumomediastinum, cervical pneumatosis, and thoracic pneumatosis, even in healthy individuals. The hypoxic and low-pressure environment of the plateau may represent an independent risk factor for the development of spontaneous mediastinal emphysema (11).

According to Boyle’s law (12), which states that as cabin pressure decreases at higher altitudes, the volume of gas inside the lungs increases. This increase in volume can potentially lead to lung injury. However, in our patient, we were unable to definitively determine the exact cause of pneumomediastinum and pneumopericardium. We hypothesize that the patient, who may have had weakened or disrupted organ barriers, experienced air leaks as a result of the altitude change during early migration to the plateau. Another possible explanation could be that the air leak was caused by the rupture of an alveolar bleb due to changes in intra-thoracic pressures at higher altitudes.

After the onset of the plateau, several factors contribute to the occurrence of spontaneous pneumomediastinum (SPM). Firstly, the pulmonary circulation is the first to react to high-altitude exposure and undergoes changes in response to acute, continuous, and chronic stages of high-altitude hypoxia. This is a crucial aspect in the development of medical issues associated with acute and chronic high-altitude exposure, as it helps the body adapt to the low-pressure hypoxic environment of the plateau. The neuro-endocrine mechanism of the pulmonary circulation is one of the earliest to undergo changes (9). Secondly, upon entering the plateau, the airway resistance and total lung resistance decrease due to the low pressure, deep and rapid breathing, and reduced turbulence. At an altitude of 3,400 m, the airway resistance can decrease by 17%. Furthermore, at an altitude of 5,000 m, there is a significant increase in maximum expiratory flow (PEF). According to the Macklin theory (13), certain inducing factors such as strenuous exercise or sudden changes in atmospheric pressure can lead to an increase in intra-alveolar pressure, causing alveolar rupture. This results in the release of air from the alveoli, which then peels off the vascular sheath and accumulates in the hilum, leading to the formation of mediastinal emphysema. If the gas pressure in the mediastinum becomes too high, the gas can diffuse along the neck, causing subcutaneous emphysema in the neck, face, and even the trunk (14, 15).

It should be acknowledged that the survey data is predominantly comprised of male migrant workers. The plateau’s working environment is notably challenging, and migrant workers are primarily involved in infrastructure projects, resulting in minimal participation from female migrant workers in the survey. Although there are a few female tourists, their representation is minimal, leading to biased data. Therefore, the occurrence of HAPE and SPM may be linked to strenuous manual labor, and there is insufficient evidence to demonstrate gender differences. In future studies, we will allocate more time to conduct a thorough and detailed classification of gender and age.

## Conclusion

By analyzing this group of cases, we have determined that CT examination is a reliable method for diagnosing early lung changes related to the plateau environment. It can also help identify complications. In addition to acute high-altitude pulmonary edema, spontaneous mediastinal emphysema (SPM) often occurs when individuals from low-altitude areas adapt to the high-altitude environment. Gas escaping to the abdominal cavity can be misdiagnosed as gastrointestinal perforation, and gas accumulation escaping into the epidural space of the spinal cord is not uncommon. The phenomenon of gas diffusion into distant tissue spaces and the mechanism of gas escape require further study. To improve the understanding and prevention of these diseases, we recommend the following preventive measures for people living in low-altitude areas (16–18): (1) People at high-altitude should avoid strenuous exercise and coughing forcefully. In cases of severe altitude sickness, appropriate oxygen therapy should be administered. (2) Stepwise adaptation is recommended. Individuals should undergo 1 week of training at an altitude of approximately 3,000 meters before ascending to higher altitudes. (3) Strengthening protection, eliminating fear, avoiding excessive mental stress, and ensuring adequate sleep are important. (4) Improving awareness of the disease is crucial. For example, chest pain should prompt immediate chest CT examination to rule out potential issues. X-ray examinations have a high rate of missed diagnosis. (5) In addition to treating the primary disease, timely mediastinal or subcutaneous drainage and decompression can relieve tension mediastinal emphysema. A comprehensive and overall treatment approach should be adopted. With early diagnosis and comprehensive treatment, a cure can be achieved. Our report provides valuable insights for the accurate diagnosis and management of spontaneous pneumomediastinum in various tissue spaces. As human activities at high-altitude increase, further research in this field is necessary.

## Data availability statement

The original contributions presented in the study are included in the article/supplementary material, further inquiries can be directed to the corresponding author.

## Ethics statement

The studies involving humans were approved by The Ethics Committee of General Hospital of Western Theater Command. The studies were conducted in accordance with the local legislation and institutional requirements. The ethics committee/institutional review board waived the requirement of written informed consent for participation from the participants or the participants’ legal guardians/next of kin because this is a retrospective observational study. Written informed consent was obtained from the individual(s) for the publication of any potentially identifiable images or data included in this article.

## Author contributions

PW: Data curation, Formal analysis, Writing – original draft, Writing – review & editing. ZZ: Formal analysis, Funding acquisition, Writing – original draft. JWu: Data curation, Investigation, Methodology, Writing – original draft. JWa: Data curation, Investigation, Methodology, Supervision, Writing – original draft. RJ: Project administration, Writing – review & editing. FD: Conceptualization, Funding acquisition, Investigation, Project administration, Resources, Supervision, Writing – review & editing.
